# Revealing latent characteristics of mobility networks with coarse-graining

**DOI:** 10.1038/s41598-019-44005-9

**Published:** 2019-05-17

**Authors:** Homayoun Hamedmoghadam, Mohsen Ramezani, Meead Saberi

**Affiliations:** 1 Monash University, Institute of Transport Studies, Melbourne, VIC 3800 Australia; 2 The University of Sydney, School of Civil Engineering, Sydney, NSW 2006 Australia; 3 University of New South Wales, School of Civil and Environmental Engineering, Sydney, NSW 2052 Australia

**Keywords:** Civil engineering, Complex networks

## Abstract

Previous theoretical and data-driven studies on urban mobility uncovered the repeating patterns in individual and collective human behavior. This paper analyzes the travel demand characteristics of mobility networks through studying a coarse-grained representation of individual trips. Building on the idea of reducing the complexity of the mobility network, we investigate the preserved spatial and temporal information in a simplified representations of large-scale origin-destination matrices derived from more than 16 million taxi trip records from New York and Chicago. We reduce the numerous individual flows on the network into four major groups, to uncover latent collective mobility patterns in those cities. The new simplified representation of the origin-destination matrices leads to categorization of trips into distinctive flow types with specific temporal and spatial properties in each city under study. Collocation of the descriptive statistics of flow types within the two cities suggests the generalizability of the proposed approach. We extract an overall displacement metric from each of the major flows to analyze the evolution of their temporal attributes. The new representation of the demand network reveals insightful properties of the mobility system which could not have been identified from the original disaggregated representation.

## Introduction

Human mobility is a significant component of urban systems. It refers to the behavior of population movements viewed as a complex system. Previous studies have discovered that both individual and collective human mobility dynamics are highly predictable^1,2^ and can be modeled accurately^3,4^. In many fields such as urban planning and public health, understanding human mobility is essential, both in its individual^5^ and collective^6^ forms and in different spatial scales^7,8^. For example, knowledge of travel patterns is crucial in epidemic control^9,10^ as it describes and predicts how infectious diseases spread in different geographical scales^11,12^. Studying intra-urban mobility is a critical step in planning and evaluation of urban development^13,14^. Furthermore, effectiveness of resource distribution projects, traffic control measures, and natural or societal disaster management plans is highly dependent on the understanding of human mobility dynamics^15–18^. Also, when analyzing the efficiency of urban infrastructure networks^19–21^, realization of patterns in human behavior from a mobility point of view is a valuable accompaniment to the knowledge on the topology of the network.

Big urban mobility data are constantly being generated by different means, including mobile phones, social media, and GPS-enabled devices. The availability of pervasive mobility data has contributed to the growing interest in studying the underlying patterns of human mobility^22–25^. Mining mobility data often leads to a profound understanding of individuals’ movements and their interaction dynamics. It also uncovers the non-trivial patterns in crowd movements or population mobility^26,27^. Many generative models are developed based on assumptions derived from known mobility pattern characteristics, to mimic the real-world transportation systems as precisely as possible and help improve the performance of existing systems^28,29^. Systems consisting of many individual elements, such as, social and computer networks, power distribution systems, and transportation systems can be modeled and studied as complex networks. This has led to several novel theoretical frameworks and applied methods offering a quantified description of real-world systems from a complex network point of view^30–32^.

Building on the growing literature of human mobility, this study shows how a coarse-grained representation of a complex travel demand network can contribute to comprehension of the spatio-temporal characteristics of the system. In this study, we develop a framework to characterize human mobility demand based on a modified special-purpose matrix coarse-graining approach^33^. We view the mobility system as a weighted directed network (see Methods) and study its characteristics through probing the summarized representation of the original large-scale form. By identifying the most influential (hotspots) source and target nodes from the rest (non-hotspots) in the network, we cluster the nodes once as origins and once as destinations. The clustering of origins and destinations is followed by dimension reduction of the mobility network. As a result, the mobility demand is represented with four major flows, (i) between hotspot origins and hotspot destinations, (ii) between non-hotspot origins and hotspot destinations, (iii) between hotspot origins and non-hotspot destinations, and (iv) between non-hotspot origins and non-hotspot destinations; we denote the proportion of trips within these flows with *HH*, *NH*, *HN*, and *NN* respectively. So, every individual flow movement on the network will be classified as one and only one of the categorized flow types. Investigating the properties of these four major indicators of the mobility reveals descriptive network-level characteristics of the system.

## Results

**Figure 1:**
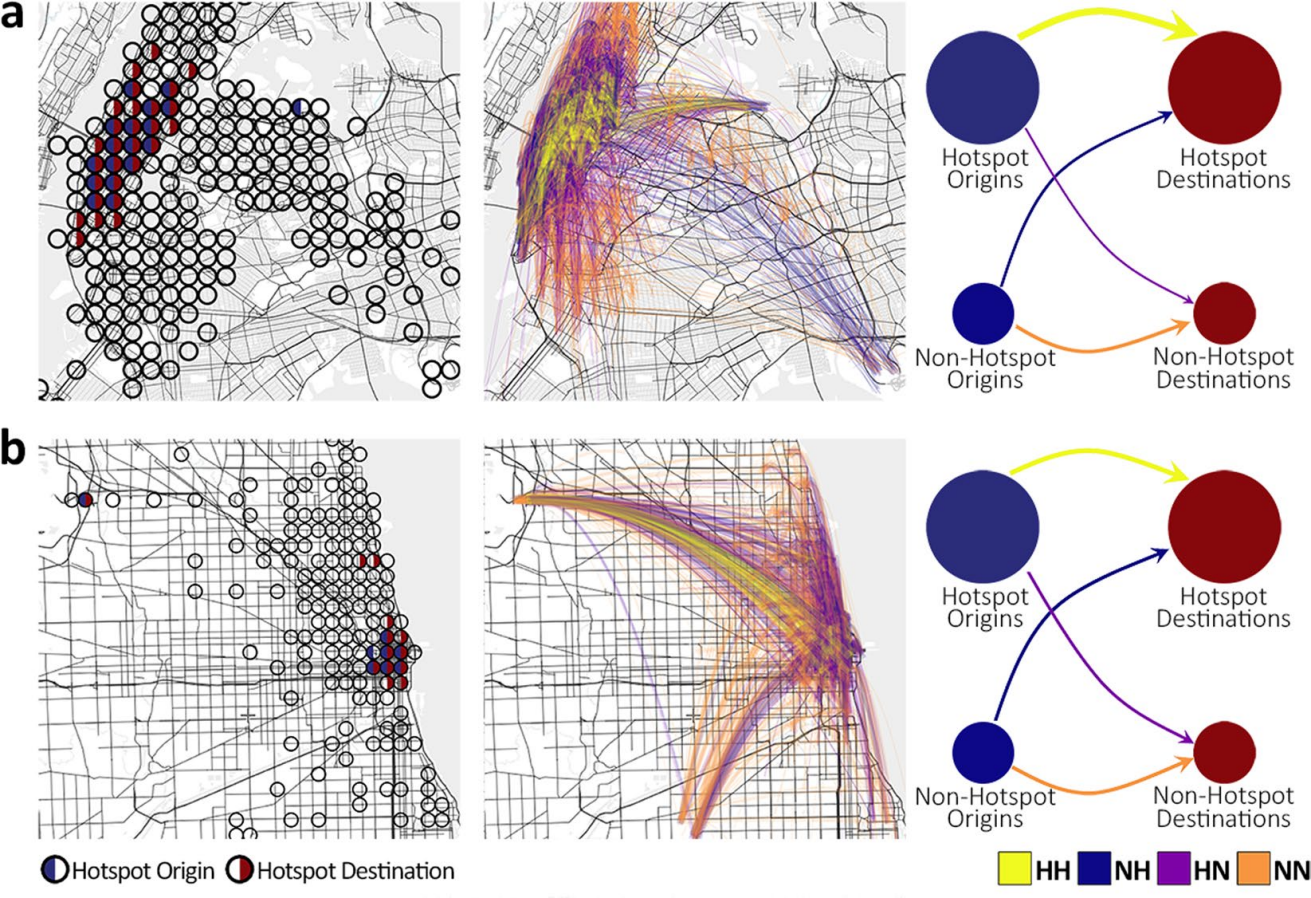
Mobility network at different granularity levels. Large-scale (middle column) and coarse-grained (right column) representation of taxi mobility data in (**a**) New York and (**b**) Chicago. Categorizing the individual flows between zones is performed based on the classification of zones into hotspots and non-hotspots once as origins and once as destinations. The distribution of the hotspots for the network of both cities is demonstrated (left column) by depicting all zones with out-going or in-coming taxi trips and whether they are identified as hotspot origins (), hotspot destinations (), both (), or none (⚪). Link widths are proportional to the number of trips associated with *HH*, *NH*, *HN*, and *NN* flows.

We generate the daily OD matrix of the taxi mobility network in Chicago and New York, and then determine the hotspot origins and destinations to describe the mobility demand with the simplified representation, i.e. the proportion of trips within each one of the four major flows. For each hour of the day, we construct a weighted directed network, where each link represents the realized travel demand between two nodes with link weighs representing the number of trips during the particular hour. Then for each hourly network, we label the links (middle column in Fig. 1) according to origin/destination hotspots (left column in Fig. 1) determined from the daily OD matrix. So, a link is labeled with exactly one flow type from *HH*, *NH*, *HN*, or *NN* based on the type of its endpoint nodes (hotspot versus non-hotspot). Proportion of each major flow is the sum of link weights labeled with the same flow type, divided by the total number of trips in the network.

### Characterization of the major flows

**Figure 2:**
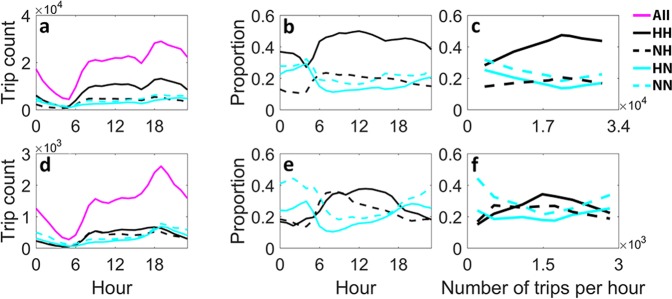
Trends in flow proportions. Temporal average number of hourly trips within different flows, and average flow proportions over all days viewed as a function of both time and total flow on the network. (**a**–**c**) Show the results for New York and (**d**–**f**) show the results for Chicago taxi mobility demand networks. (**a**,**d**) Show the evolution of number of trips corresponding to each flow type throughout the day. In (**b**,**e**) *HH*, *NH*, *HN*, and *NN* time series are depicted for New York and Chicago respectively. Flow proportions in (**c**) New York and (**f**) Chicago are seen as a function of total number of trips per hour on the mobility network; curves are generated with data points calculated through equal frequency binning.

The number of trips associated with each flow type varies temporally throughout the day with a positive correlation with the total number of trips; see Fig. 2a for New York and Fig. 2d for Chicago. In order to analyze the temporal evolution in hourly *HH*, *NH*, *HN*, and *NN* flow proportions, we take the average of proportions over 28 days of February 2015; Fig. 2b (New York) and Fig. 2e (Chicago) depict average flow proportion time series with their temporal inter-correlation.

Flow proportions sustain a strong inter-correlation as they vary throughout the day, which is observed in both cities. Considering *HH*-*NH* and *HN*-*NN* paired together, strong positive correlation is observed within each pair and negative correlation between any two flow types selected from different pairs. To quantitatively assess the correlations between these variables, we calculate the Pearson product-moment correlation coefficient between all possible pairs of flow types as shown in Table 1. Although *HH* flow constitutes the largest proportion of the trips for almost all day in New York, *HN* and *NN* in Chicago overtake the dominance of *HH* for almost half of the day (Fig. 2e); *HN* and *NN* are dominant flows in Chicago starting right after 18:00 until early morning hours when the number of hourly trips in the network is at its minimum or maximum (compare Fig. 2e and f). Note that although the number of trips within each flow type have similar temporal patterns (positively correlated with the total flow), proportional flow values show an alternative interpretation of the mobility network with similar properties repeated in both cities, manifesting the transformation made by coarse-grained representation of the mobility network.

**Table 1:** Correlation among temporal flow proportions. Pearson correlation coefficients between all pairs of flow types. Each entry shows Pearson’s *r* between hourly time series of two flow types for New York (left panel) and Chicago (right panel).

	New York				Chicago			
Flow Type	*HH*	*NH*	*HN*	*NN*	*HH*	*NH*	*HN*	*NN*
*HH*	1	0.81	−0.94	−0.96	1	0.77	−0.75	−0.99
*NH*	0.81	1	−0.94	−0.88	0.77	1	−0.97	−0.80
*HN*	−0.94	−0.94	1	0.92	−0.75	−0.97	1	0.74
*NN*	−0.96	−0.88	0.92	1	−0.99	−0.80	0.74	1

Here, we investigate the trends in the flow proportions as a function of total number of trips over the network; see Fig. 2c and f. With increasing number of trips on the network, *HH* and *NH* proportions rise until they reach their peak, approximately at the same value of total flow, and then they decline until hourly load reaches its maximum. However, an opposite pattern is observed for *HN* and *NN*. Surprisingly, with growing number of trips per hour in the taxi mobility network, the increase-then-decrease behavior of *HH* and *NH*, and decrease-then-increase in *HN* and *NN* proportions, are observed in both New York and Chicago, despite the fundamental differences between the two cities and their networks.

Demand component *HH* can be interpreted as the flow between the critical locations in the network such as within the central business district (CBD) or between the CBD and other areas with relatively high attraction (e.g. dense residential areas and airports). Originated from numerous non-hotspot zones, *NH* targets the relatively few hotspot destination zones in the city; *HN* does the opposite moves passengers from critical zones to the often insignificant destinations. Commuting trips in a metropolitan area can be constituent of different types of major flows depending on the land use intertwined with travel behavior. The temporal evolution of major flow proportions (Fig. 2a and d) with revealed information on temporal magnitude of demand (Fig. 2b and e) demonstrate the similarities and distinctions between constitutions of major flows in different cities. For example the increase in *NH* for both cities during the morning peak period shows the contribution of home-to-work trips to this flow in both cities, or the growth in *HN* for Chicago but not for New York during the evening peak manifests the difference between the work-to-home trip dynamics of the two cities.

**Figure 3:**
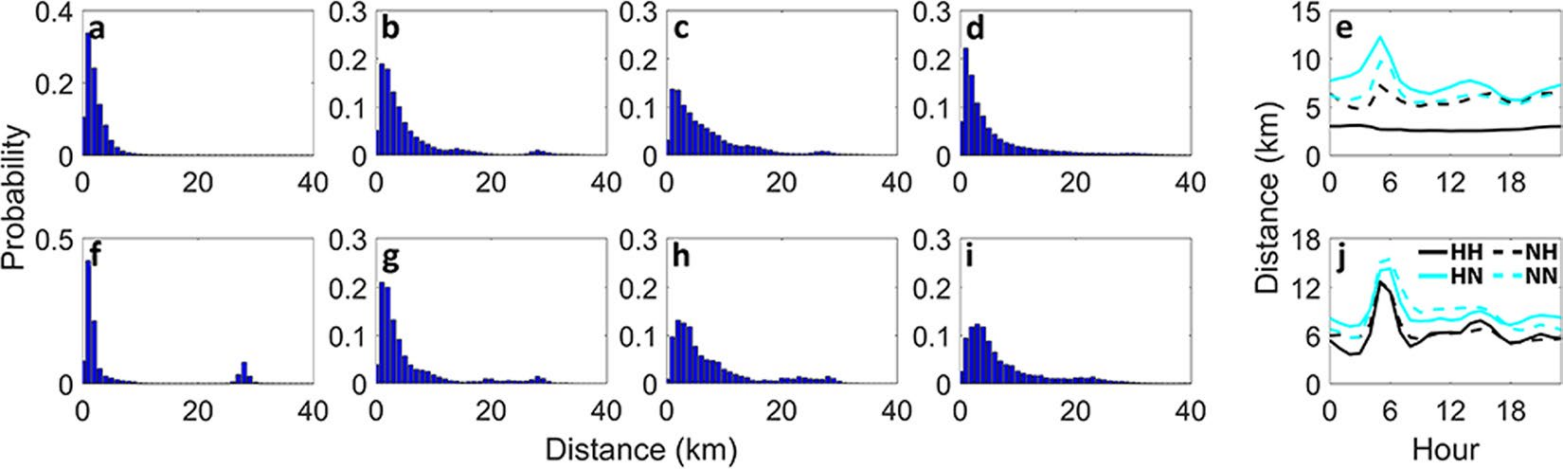
Travel distance characteristics of major flow types. Trip distance distribution and trip distance time series of *HH*, *NH*, *HN*, and *NN* flow types during the whole month of February 2015 for New York (first row) and Chicago (second row). In the left panel, from left to right trip distance distributions are corresponding to (**a**,**f**) *HH*, (**b**,**g**) *NH*, (**c**,**h**) *HN*, and (**d**,**i**) *NN* flow types. Hourly trip distance time series averaged over all days included in the available data, is depicted for (**e**) New York and (**j**) Chicago.

**Figure 4:**
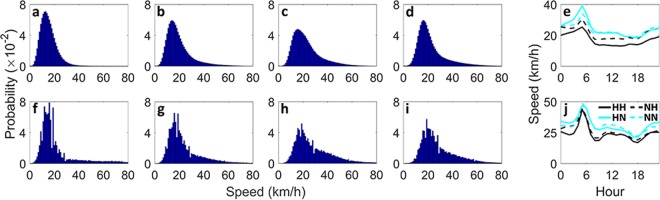
Travel speed characteristics of major flow types. Trip speed distribution and hourly average trip speed of flow types for New York (first row) and Chicago (second row). In the left panel, from left to right trip speed distributions correspond to (**a**,**f**) *HH*, (**b**,**g**) *NH*, (**c**,**h**) *HN*, and (**d**,**i**) *NN* flow types.

Furthermore, we explore the distribution of travel distance and speed (Figs 3 and 4) for the identified major flows. We observed that different flow types show different characteristics within each city while each flow type has similar characteristics across the two cities. For both New York and Chicago, the trip distance distribution for *HH* shows a small variance and a rapid decay in frequency with increasing distance compared to the other flow types (Fig. 3a and f). This is expected as the most significant (hotspot) nodes in the networks are often clustered together spatially, which is often the case in mono-centric cities. However, a small peak approximately at 28 km for Chicago (Fig. 3f) reveals the existence of a significant zone at that distance from the CBD which is the O’Hare International Airport in this case. Nevertheless, in New York, only one of the airports (LaGuardia Airport) is classified as hotspots and that is for a subset of days, which is due to the relatively high travel demand to and from Manhattan area and also the existence of other preferred means of transportation over taxi to and from the airports. Furthermore, these airports have multiple pick-up/drop-off points which are associated with more than one node in the network, hence the demand associated with the airport is not concentrated as one might expect.

The distribution of trip distance for *NH* in both cities (Fig. 3b and g) has a slower frequency decay with increasing distance, when compared to *HH*. For *HN*, the travel distance distribution has even a longer tail (Fig. 3c and h), suggesting higher number of trips with larger distances when traveling from important centers (hotspots) to other areas (non-hotspots). Hourly average trip distances for different flow types in New York and Chicago also reveal that all flow types have a peak in travel distance just before the start of the morning peak, except for *HH* in New York which remains roughly invariant throughout the whole day. Despite the observed differences in trip distance distributions for different flow types, consistent and common temporal characteristics exist between the same major flow types in the two cities.

The distributions of travel speeds also show a discrepancy among different major flow types within each city and similar characteristics for each flow type across different cities; see Fig. 4. The noise observed in the distributions from Chicago can be a result of abundant missing values within the raw data. The variance in journey speeds increases respectively for *HH*, *NH*, and then *HN* flows. The most frequent trip speeds move toward higher speed values with a decrease in the largest frequencies comparing *HH*, *NH*, and *HN* flows respectively (Fig. 4a–c and f–h). Despite the differences between trip speed distributions, flow types have similar temporal behavior in both cities; with a peak in the beginning of the morning and lower values with relatively low variation throughout the day (see Fig. 4e and j). In both cities, there is a strong negative correlation between the temporal evolution of speeds associated with different major flows and the total flow on the network. Reduction in the total flow, generally leads to less congested road network and this increases the average speed of taxi trips. This is consistent with the patterns observed for the average distance associated with each flow. The average distance of the trips in almost all flows (other than *HH* flow in New York) increases with the growing average speed. This suggests that with the drop in total flow on the network which enables the road network to accommodate higher speed taxi trips, passengers become more inclined to use the taxi mode for their long-distance trips. In both cities, we observe similar temporal patterns for the average speed, which is consistent and also correlated with what is observed for travel distance. For New York, most of the hotspot zones are located in Manhattan area (see Fig. 1), so the *HH* flow in New York is often circulating a relatively small area which tends to be congested during the day, hence the average distance of trips in this case remains temporally stable.

### Repeating patterns in flow displacement

Each taxi trip makes a displacement by changing the position of the users. The displacement of a trip can be described by a vector with the origin at the pick-up location pointing to the drop-off location (the endpoint of the trip). Here, we explore the major travel flows *HH*, *NH*, *HN*, and *NN* to characterize them in terms of the displacement they make in the mobility system. To study the overall displacement for different major flow types, we calculate the displacement vector of each flow type in every hour interval of the day. For each flow type *f* ∈ {*HH*, *NH*, *HN*, *NN*}, following calculations are performed for each hourly network. For each link *e* in the network we denote the number of trips over that link with a link weight *w*_*e*_ and the average distance of the trips corresponding to that link with *d*_*e*_, so the overall displacement vector for each flow type *f* can be expressed as

1 $${\overrightarrow{V}}_{f}=(\sum _{\{e|{t}_{e}=f\}}{d}_{e}{w}_{e}{x}_{e},\sum _{\{e|{t}_{e}=f\}}{d}_{e}{w}_{e}{y}_{e})$$

where *t*_*e*_ is the determined label for the link *e* indicating the major flow it belongs to, and *x*_*e*_ and *y*_*e*_ are the distance between locations of the link endpoints along east and north respectively. In other words, if we assume that the study area is projected on a Cartesian plane with y-axis along geographical meridian and pointing toward north, *x*_*e*_ and *y*_*e*_ represent the displacement toward east and north respectively, by traveling along the link *e*. In order to characterize the displacements made by the flows, we study the evolution of angle *θ* and magnitude *r* of the displacement vector over time. Angle and magnitude are derived from the overall flow displacement vector as

2 $${r}_{f}=|{\overrightarrow{V}}_{f}|=\sqrt{{(\sum _{\{e|{t}_{e}=f\}}{d}_{e}{w}_{e}{x}_{e})}^{2}+{(\sum _{\{e|{t}_{e}=f\}}{d}_{e}{w}_{e}{y}_{e})}^{2}}$$

3 $${\theta }_{f}=\measuredangle {\overrightarrow{V}}_{f}=catan\,(\sum _{\{e|{t}_{e}=f\}}{d}_{e}{w}_{e}{x}_{e},\sum _{\{e|{t}_{e}=f\}}{d}_{e}{w}_{e}{y}_{e})$$

where *catan* is a customized multi-valued variation of the standard inverse tangent function with the output angle value *catan*(.,.) ∈ (0, 2*π*]; definition of *catan* function can be found in Section 4 of SI. Overall displacement angle and magnitude are spatial attributes of the major flows which are time-varying as expected. Calculation of these attributes for major flows allows us to analyze the temporal evolution of spatial characteristics of the derived major flows on the mobility network.

**Figure 5:**
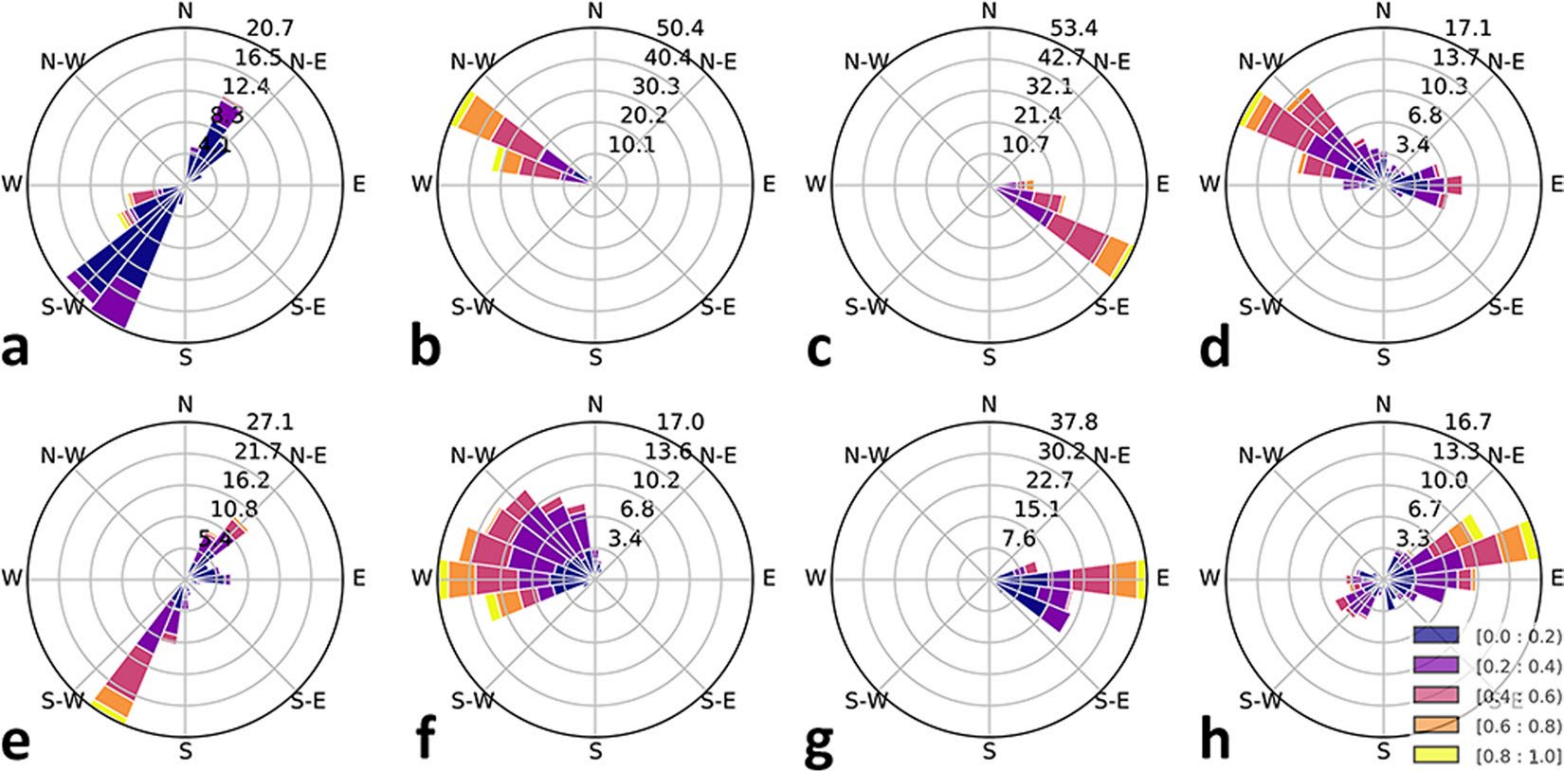
Direction distribution of major flows displacement. Each plot visualizes the distribution of angle and magnitude of overall displacement associated with major flow types. Top row shows the results for New York and bottom row corresponds to Chicago with plots corresponding to (**a**,**e**) *HH*, (**b**,**f**) *NH*, (**c**,**g**) *HN*, and (**d**,**h**) *NN* flows. Proportion of trips in each direction is represented by the length of polar bar with respect to the percentages shown on the concentric circles. Also for each direction, the distribution of displacement magnitudes are demonstrated by sectioning the corresponding bar; section length shows the frequency of a magnitude and section color corresponds to a particular range of magnitude values, while the displacement magnitudes are scaled between 0 and 1.

**Figure 6:**
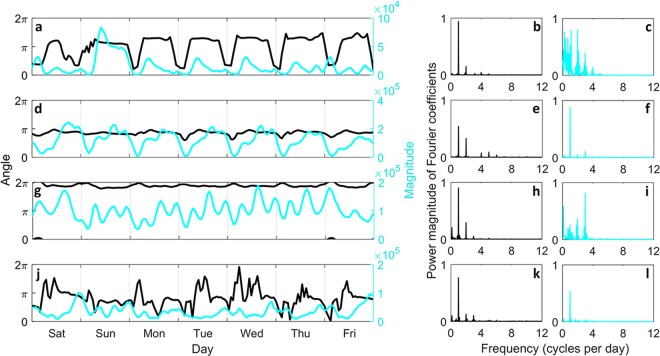
Displacement analysis of major flows in New York taxi mobility network. Left panel shows the time series of hourly angle and magnitude of overall displacement for (**a**) *HH*, (**d**) *NH*, (**g**) *HN*, and (**j**) *NN* flow, during the last week of February 2015. For a closer look at the *NH* and *HN* flow proportion time series, see Fig. S5 in SI. Right panel shows the scaled magnitude of DFT coefficients also called periodogram for (**b**,**c**) *HH*, (**e**,**f**) *NH*, (**h**,**i**) *HN*, and (**k**,**l**) *NN* flow with the first column (**b**,**e**,**h**,**k**) presenting the results for displacement angle time series and the second column (**c**,**f**,**i**,**l**) showing the results for displacement magnitude time series. The magnitude of the DFT coefficients are normalized for each periodogram to have the same scale in the range of values between 0 and 1.

**Figure 7:**
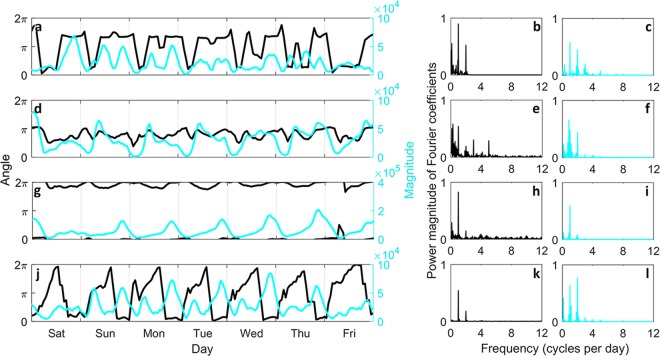
Displacement analysis of major flows in Chicago taxi mobility network. Left panel shows the time series of hourly angle and magnitude of overall displacement for (**a**) *HH*, (**d**) *NH*, (**g**) *HN*, and (**j**) *NN* flow, during the last week of February 2015. The right panel shows the scaled magnitude of DFT coefficients for (**b**,**c**) *HH*, (**e**,**f**) *NH*, (**h**,**i**) *HN*, and (**k**,**l**) *NN* flow with the first column (**b**,**e**,**h**,**k**) demonstrating power of Fourier coefficients for temporal displacement angle and the second column (**c**,**f**,**i**,**l**) showing the power of periodic components in temporal displacement magnitude.

Now, for each hourly generated network, with its edges labeled as *HH*, *NH*, *HN*, or *NN*, *r*_*f*_ and *θ*_*f*_ is calculated; see Fig. 5 for the distributions of angle and magnitude of overall displacements. We investigate the time series of each variable, i.e. *r*_*f*_ and *θ*_*f*_, for each major flow type; see Fig. 6 for New York and Fig. 7 for Chicago. It is observed that a periodic pattern exists in most of the time series, particularly a daily pattern. A classic approach to numerically quantify the periodicity in a time series, is discrete Fourier transform (DFT) based spectral analysis through modulus of the DFT coefficients^34^. We calculate the periodogram (see Methods for details) of angle and magnitude of overall displacement time series for New York and Chicago which are depicted in Figs 6 and 7, respectively. Periodogram shows the relative presence of periodic signals at different frequencies.

Analyzing overall displacement vectors of major flows, reveals latent characteristics of the mobility network. The displacement of each flow shows distinctive features with temporal periodic patterns. In New York, the overall displacement of *HH* flow is directed toward southwest from early morning to until a few hours before midnight, during which the magnitude decreases almost consistently. Displacement of *HH*, then faces the opposite direction for the remaining hours of the day (Fig. 6a). Interestingly, these two opposite directions are both along the general topography of Manhattan, and with significant number of hotspots being inside Manhattan area, this shows how the topographic structure of the urban area can be manifested in behavior of a major flow. During the day, *NH* (Fig. 6d) and *HN* (Fig. 6g) major flows make an overall displacement always toward west-northwest and east-southeast, respectively. These directions are in fact the directions inward and outward of Manhattan. Nonetheless, *NH* and *HN* displacement magnitudes, show a unique temporal pattern and periodicity. *NN* flow does not show a clear pattern, however, a significant periodic component with one cycle per day exists in both angle and magnitude signals, according to periodograms of this flow type in New York; see Fig. 6k and l for the periodograms calculated for the angle and magnitude time series of the *NN* flow displacement.

The angle time series of all flows have similar periodicity, as in the periodograms the most significant component by far is observed for one cycle per day frequency. However, the daily pattern in the angle of *NH* flow displacement is not as significant as the other three flows and there exists another component with two cycles per day (Fig. 6e). Magnitude of overall displacement vector has a significant daily pattern for *NH* flow (Fig. 6f) and also for *NN* flow (Fig. 6l). For *HN* flow the most significant periodic component is repeated three times per day and the second strongest component demonstrates that there is also a weekly pattern in the temporal evolution in magnitude of the *HN* flow displacement; see Fig. 6i. The periodogram of *HH* displacement magnitude is more complex comparatively. A large number of high-power components with different frequencies makes it infeasible to conclude about the periodic patterns within the signal, merely based on the periodogram.

For Chicago network, the overall displacement of *HH* flow is directed approximately toward northeast during morning and evening peak hours, whereas it points toward south and southwest for most of the remaining hours of the day (Fig. 7a). The magnitude of overall displacement vector is relatively small during peak hours, meaning that the constituent trips of the *HH* flow are not much concurrent. This can be explained if the starting points of trips toward the CBD during the morning peak and endpoints of trips going back from CBD during the evening peak, are scattered over the whole metropolitan area. Yet, it is interesting that still the overall displacement direction has a repeating daily pattern, given all the irregularities associated with the trips constituting the *HH* flow. Angle (Fig. 7b) and magnitude (Fig. 7c) periodograms of the overall *HH* displacement suggest the existence of a periodic component with one cycle per day. The second major periodic component of the displacement angle for the *HH* flow, has a frequency of one cycle per week as it is seen in Fig. 7b with the high power magnitude of Fourier coefficient corresponding to that frequency.

Displacement angle of the *NH* flow varies between north and west during the day (Fig. 7d), while the displacement magnitude is relatively small when the flow is facing north. Existence of a significant periodic pattern over time cannot be asserted for the angle time series based on the periodogram in Fig. 7e. However, the overall displacement magnitude of the *NH* flow has a clear daily pattern, and can be deduced quantitatively from the largest power of magnitude which is associated with the frequency of one cycle per day in the periodogram shown in Fig. 7f.

The magnitude of the overall *NH* displacement has its highest values during morning peak period when the *NH* flow is going approximately toward west, but for the *HN* flow the opposite is seen as the magnitude is highest around evening peak hours with the angle pointing toward east (Fig. 7d and g). Displacement vector of *HN* flow has an overall angle facing east and southeast throughout the day with tendency toward east when the magnitude of the vector has higher values (Fig. 7g). Both angle and magnitude of the overall displacement vector have a clear daily pattern according to their preiodograms in Fig. 7h and i, respectively. *NN* flow displacement in Chicago has a daily pattern although the angle covers almost the whole range of possible directions (Fig. 7j). Spectral analysis suggests that a daily pattern is the most powerful periodic component of the angle signal (Fig. 7k) and there exists a periodic component within the magnitude time series with repeating in two cycles per day (Fig. 7l).

## Discussion

The paper has presented a method for dimension reduction of fine-grained mobility demand network. We modified a previously proposed coarse-graining method^35^ and applied it to taxi mobility data from New York and Chicago. We used a non-parametric clustering algorithm to separate nodes into hotspot and non-hotspot classes as origins and as destination; based on their significance in the mobility networks. The presented coarse-graining method produces a simple 2 × 2 matrix from the initial OD matrix, representing the proportion of four conceptually distinctive major flows, namely, hotspot origin to hotspot destination (*HH*), non-hotspot origin to hotspot destination (*NH*), hotspot origin to non-hotspot destination (*HN*), and non-hotspot origin to non-hotspot destination (*NN*). We showed that each resulting flow type has different characteristics with a unique temporal signature throughout the day. While each flow type behaves differently when compared to one another, the same flow types have similar characteristics across the two cities under study, despite the fact that these cities are different in structure and topography. We also introduced an overall displacement feature for each extracted flow type, and demonstrated the existence of periodic patterns within each flow. We used DFT to quantify the patterns and to shed light on the latent collective dynamics within the mobility network that could not have been discovered from the original representation of the system without coarse-graining.

Assuming taxi mobility is a reasonable proxy to general urban mobility, the quantified patterns can help with more efficient transportation service network design and validation of estimated origin-destination demand matrices from both aggregated and disaggregated travel demand models. New insights on the collective behavior of classes of trips in a city, measured by displacement, can also be leveraged for optimized travel demand management, better public transport service design, improved matching mechanisms in ride-sharing systems, and potentially in efficient transportation pricing.

A limitation to this study is that the analyzed mobility data is restricted in scope and there is no capacity to further investigate the movement pattern in terms of causes and the effects. Incorporating additional information such as temporal social network data to study the interdependency between the mobility and social networks^36,37^ can be a new direction for future analysis in accordance to the proposed framework. The proposed framework, can be integrated with transportation network models, whether through utilizing the modified coarse-graining method or leveraging our findings to decipher the demand characteristics. The only input to the proposed framework is the OD demand matrix of a mobility system, so the framework is generalizable to other travel modes and also can be adopted to study a multi-modal system as it does not take any assumption on the specifications of the subject mobility system. Consequently, another potential aspect to be further investigated is to apply the proposed framework to other mobility systems with different services and structure.

## Methods

### Data description and OD matrix generation

We use openly available and widely used datasets of taxi trip records from New York and Chicago. Each record in the data holds the information from a single taxi trip, collected automatically by on-vehicle devices or manually by taxi driver, with a number of attributes including a timestamp, geographical coordinates of the pick-up/drop-off points, and the trip distance. An average speed for each trip can be calculated using the trip distance and duration.

We use the data collected during February 2015 in both cities. A comprehensive data cleaning process is performed as the first step to remove the erroneous records and those with non-derivable missing values. Records with a trip distance less than the Euclidean distance between pick-up and drop-off locations and also records with a trip distance less than 300 m are deemed invalid and removed from the data. Trips with duration less than 60 sec or average speed over 120 km/h are also eliminated from the dataset. We further clean the data by removing trips with travel distance being three times larger than the Manhattan distance between pick-up and drop-off locations; we consider these trips as misinforming due to the possibility that multiple trips with different purposes are aggregated into one record. The original Chicago and New York taxi data during February 2015 include a total of 2,303,627 and 13,990,385 records, respectively. After cleaning, the data consist of 954,745 valid records for Chicago and 12,617,928 valid records for New York; a large number of Chicago taxi trip records are invalid merely due to missing pick-up/drop-off geographical coordinates.

We use a grid with 1 km^2^ cells to generate the initial OD matrix of the mobility system within which each entry showing the number of trips from the grid cell associated with the row ro the grid cell associated with the column; the matrix is of size *n* which is the number of grid cells with at least one in-coming or out-going trip. Also, a network structure corresponding to the grid is constructed, where each node corresponds to a grid cell and there is a directed link from node *i* to node *j*, if there is at least one trip between the corresponding cells. We verified that the output of our coarse graining method is independent of the spatial aggregation size used to generate the OD matrix (see Fig. S3). We investigated a set of preliminary results for networks generated based on different grid sizes corresponding to the square side lengths of 0.5, 1, and 2 kilometers and observed no significant difference in the final results (see Section 2 in SI).

### Coarse-graining of the  OD matrix

Our methodology for characterizing mobility networks is built upon a previously proposed coarse-graining approach^33,35^. The idea is to reduce the complexity and dimensionality of a large-scale mobility network (see SI section 3) while trying to preserve meaningful properties of the original network to a possible extent. The method presented in^33,35^ reduces a large-scale origin-destination (OD) matrix to a 2 × 2 matrix by categorizing nodes as hotspots and non-hotspots based on the number of trip endpoints to/from each node. A node, participates in the classification process once as an origin and once as a destination. Then four possible flow types within the network are determined as (i) from hotspot origins to hotspot destinations, (ii) from non-hotspot origins to hotspot destinations, (iii) from hotspot origins to non-hotspot destinations, and (iv) from non-hotspot origins to non-hotspot destinations which we denote *HH*, *NH*, *HN*, and *NN* flow respectively. To aggregate the cells corresponding to each one of the four possible flows, the cells are summed up and normalized by the total number of trips in the network. This can simply be achieved by sorting both rows (higher values toward the top) and columns (higher values toward the left) of the OD matrix separately according to their sum, i.e. total number of out-going or in-coming trips for each node, and then partitioning the OD demand matrix into four sub-matrices by separating hotspots from non-hotspots, once for matrix rows (origins) and once for columns (destinations). The coarse-grained matrix can be expressed as

4 $${\rm{\Lambda }}=[\begin{array}{cc}HH & NH\\ HN & NN\end{array}]$$

where its elements are calculated according to the original large-scale OD matrix *F* as below:

5 $$HH=\sum _{i\in M,j\in P}{F}_{ij}/\sum _{i,j}{F}_{ij}$$

6 $$NH=\sum _{i\notin M,j\in P}{F}_{ij}/\sum _{i,j}{F}_{ij}$$

7 $$HN=\sum _{i\in M,j\notin P}{F}_{ij}/\sum _{i,j}{F}_{ij}$$

8 $$NN=\sum _{i\notin M,j\notin P}{F}_{ij}/\sum _{i,j}{F}_{ij}$$

where *M* (*P*) is hotspot origin (destination) set. Hotspot set can be defined as the set of origins (destinations) with number of outgoing (incoming) trips higher than a determined threshold or can be formed with the top *c* origins (destination) with largest number of outgoing (incoming) trips. Intuitively, we have *HH*, *NH*, *HN*, *NN* ∈ [0, 1] and the sum of all entries of the matrix Λ is equal to 1. The coarse-grained form of OD demand matrix (Λ) represents the network with the proportions of four major flow types circulating the mobility system.

### Node clustering

Our modification to the coarse-graining method of Louail *et al*.^33,35^ lies in the node clustering stage. The four major flows types, *HH*, *NH*, *HN*, and *NN* are derived from a large-scale OD matrix as a result of a two-class clustering once for origins and once for destinations to determine hotspot nodes.

In order to separate hotspot and non-hotspot nodes, we use a centroid-based clustering method, which breaks a sorted list of scalars into two classes of higher and lower values (also see Section 1 in SI). First, we sort node flux-out (flux-in) values, which are row (column) sums of  the initial mobility OD matrix. For *n* sorted row (column) sums as *q*_1_ > *q*_2_ > … > *q*_*n*_, we use the clustering algorithm to find the separation point *c*_*o*_ (*c*_*d*_) determining the number of hotspot origins (destinations) corresponding to the *c*_*o*_ (*c*_*d*_) largest flux-out (flux-in) values, i.e. ${q}_{1},{q}_{2},\ldots ,{q}_{{c}_{o}}({q}_{1},{q}_{2},\ldots ,{q}_{{c}_{d}})$. The separation point *c,* can be found as described below:

9 $$\mathop{{\rm{\arg }}\,{\rm{\min }}}\limits_{c}\,\sum _{i\mathrm{=1}}^{c}|{q}_{i}-\frac{1}{c}(\sum _{k\mathrm{=1}}^{c}{q}_{k})|+\sum _{j=c+1}^{n}|{q}_{j}-\frac{1}{n-c}(\sum _{l=c+1}^{n}{q}_{l})|$$

where *q*_*i*_ can be either the sum of a row or a column from the mobility OD matrix. Solving Equation (9) for sorted list of row sums results in *c*_*o*_ and for the sorted list of column sums, results in *c*_*d*_. We then use the estimated thresholds to categorize the nodes, into hotspots and non-hotspots.

### Periodogram

Having a series *x*_1_, *x*_2_, …, *x*_*N*_ sampled at the frequency *f*_*s*_ = 1/Δ*t*, the periodogram *P*_*k*_, *k* = 0, 1, …, *N*/2 can be calculated as the magnitude of DFT coefficients at Fourier frequencies of *f*_*k*_ = *kf*_*s*_/*N*, *k* = 0, 1, …, *N*/2. Periodogram shows the relative presence of periodic signals at different frequencies.

We first apply a zero-mean normalization to the time series with *N* = 672 during a month interval which makes the sampling frequency of *f*_*s*_ equal to 24 per day. We then calculate the periodogram of each time series for Fourier (fundamental) frequencies spanning from 0 to 12 cycles per day, at steps with distance of *f*_*s*_/*N* = 24/672 = 1/28. The periodogram is then scaled so the magnitudes can be illustrated within the range [0, 1].

## Supplementary information

Supplementary Information

## Data Availability

The datasets analyzed in this study are publicly available from the New York City Taxi Limousine Commission accessible via  https://www1.nyc.gov/site/tlc/about/tlc-trip-record-data.page https://www1.nyc.gov/site/tlc/about/tlc-trip-record-data.page (Access Date: 11/03/2019), and from the City of Chicago via  https://data.cityofchicago.org/Transportation/Taxi-Trips/wrvz-psew/data (Access Date: 11/03/2019).
