# Supplementary material for: Revealing latent characteristics of mobility networks with coarse-graining
Source: Sci Rep. 2019 May 17;9:7545. doi: 10.1038/s41598-019-44005-9 (PMC6525175; doi:10.1038/s41598-019-44005-9)
Supplement: Supplementary file 1 — Supplementary Information [file 41598_2019_44005_MOESM1_ESM.pdf]

Supplementary Information for

Revealing latent characteristics of mobility networks with coarse-graining

Homayoun Hamedmoghadam, Mohsen Ramezani, and Meead Saberi

1. Sensitivity of the clustering methods to variation in the travel demand

We generate the Origin-Destination (OD) travel demand matrix of a mobility network, within which each row (column) corresponds to a node in the network as an origin (destination) and each entry of the matrix quantifies the number of trips from corresponding origin to destination. We perform the coarse-graining of the large-scale OD matrix through a two-class clustering of sorted row sum and column sum values, each leading to separation of nodes into hotspots from non-hotspots as origins (rows) and destinations, respectively. Consider a set of *n* sorted values $q_1 > q_2 > \ldots > q_n$ corresponding to the in (or out) flux for each node in the mobility network. A threshold c with $1 < c \leq n$ determines hotspot nodes which are the nodes associated with the flux values equal or greater than q_c. Our work is motivated by a previous study proposing the extraction of an OD matrix signature using a method called LouBar to find the threshold *c*; see [1, 2] for details. LouBar determines a separation point *δ* between hotspots and non-hotspots, with $c = \lfloor n\delta \rfloor$, calculated according to the Lorenz curve of the distribution of the node fluxes using the tangent of the curve at the point where the curve ends, i.e. always the point (1, 1). The separation point *δ* is calculated as below:

$$\delta = \frac{\sum_{i=1}^{n} q_i}{n q_1} \tag{1}$$

Now assume a change in destination (origin) for a number of trips *d* to the node with the largest in-flux (out-flux), which is associated with q_1 trips. Let the new distribution of flux values be described as $q_1 + d = q'_n > q'_2 > \ldots > q'_n$, where $q'_i, i = 1, \ldots, n$ is the new flux value for node i after changes are applied. The separation point will move toward nodes with larger flux by a magnitude that can be expressed as follows, independent from the new flux values:

$$\Delta\delta = \frac{d \sum_{i=1}^{n} q_i}{n(q_1^2 + d q_1)} \tag{2}$$

Looking at Eq. (2), one can see that the change in the threshold depends on *d* (number of altered trips) rather than the nodes from which that flux is deducted. Also, any further change to the trip endpoints between the rest of the nodes, if leaves the largest value $q'_n = q_1 + d$ unchanged, has no impact on the threshold found by LouBar; even if it changes the order of node fluxes significantly. We argue that sensitivity of LouBar method to the largest flux value has adverse effect on determination of hotspots.

Here, simulations illustrate the effect of change to trip endpoints according to two different scenarios, on the number of hotspots identified by LouBar and our proposed method. Simulation of altering the trip end points is initiated with the actual daily OD matrix of the network and follows a scenario to manipulate the initial OD matrix. In the first simulated scenario, trip destinations (origins) are altered from a non-hotspot node to the node with highest flux-in (flux-out). Decreasing the flux of non-hotspots as one expects does not change the label of those nodes, but with increasing flux of the first ranked node the hotspots with the smallest flux might become too far from the hotspot class average. This has a small impact on the number of hotspots determined by our method (see Fig. S1). However, LouBar is too sensitive to the changes in the flux of the first ranked node, and as the first ranked nodes' flux increases the number of hotspots decrease almost linearly dependent on the number of altered trip endpoints. In the second simulated scenario, trip endpoints are selected from the hotspot nodes other than the first ranked node and altered to a non-hotspot node (see Fig. S2). Intuitively, one can predict that decrease in hotspots flux and increase in non-hotspots flux should definitely have an impact on the number of determined hotspots. This however only happens when we apply our proposed method to the OD matrix, while the number of hotspots returned by LouBar method remains constant as the flux of the first ranked node remains intact and the sum of all other fluxes are fixed too.

2. Size of the grid cells for zoning

We perform our proposed coarse graining approach on different OD matrices generated based on grids with different cell sizes to investigate whether the results depend on the grid cell size or not. Fig. S3 shows the calculated flow proportions over time for three taxi mobility demand networks built from taxi trip data in New York based on different zoning corresponding to different grid lengths. As can be seen, the time series of each flow type look very similar between different networks, with temporal trends and inter-correlations being almost the same as well as the calculated proportion of each flow type over time. Therefore, we argue that the observed patterns are independent of the size of the grid.

3. Complexity reduction by the coarse-grained representation

Here we present an example to demonstrate the extent of reduction in complexity of the original OD demand matrix made by the coarse-graining method. Fig. S4 visualizes the OD matrix of taxi mobility system in New York during one hour. The matrix includes every node with at least one out-going or in-coming taxi trip. Nodes are representative of 1 km^2 square zones and they are numbered according to their geographical positioning from west to east starting from the row of zones in the north, and then row by row toward south. In the example depicted in Fig. S4.a, the network size is 569 with 5889 directed links, meaning the network density (number of links divided by the number of possible links) is $D = 0.018$ which relatively

low although size of the corresponding OD matrix is large. By applying the coarse-graining approach with the introduced clustering method, the matrix of size 569×569 with 5889 non-zero entries will be reduced to a 2×2 matrix. This serves our purpose of a great extent of reduction to 4 major flow in the network which are intuitive and interpretable.

4. *Customized inverse tangent function*

In order to find the angle between a vector (x, y) and the positive x-axis, we define a customized multi-valued function $catan(.,.)$ to adjust the standard *arctan* function so it returns an angle value in the range $(0, 2\pi]$. Function $catan(.,.)$ is defined as follows:

$$catan = \begin{cases} \arctan\left(\frac{x}{y}\right) + \pi & x < 0 \\ \arctan\left(\frac{x}{y}\right) & x > 0, y \geq 0 \\ \arctan\left(\frac{x}{y}\right) + 2\pi & x > 0, y < 0 \\ \frac{3\pi}{2} & x = 0, y < 0 \\ \frac{\pi}{2} & x = 0, y > 0 \\ undefined & x, y = 0 \end{cases} \tag{3}$$

5. *Periodicity in angle timeseries*

Fig. 6 of the manuscript represents the angle time series of the displacement vectors in a fixed range of degrees between 0° and 360°. Here, the angle time series of the overall displacement vectors for *NH* and *HN* flows in New York are plotted (see Fig. S5) in a narrowed range of degrees over time for better visualization and comparison; the periodicity of the pattern is more perceptible in the enlarged plot.

6. *Closer look at NH and HN flows in Chicago*

Given that Chicago's CBD is located on the east side of the metropolitan area, one might expect the overall displacement vector of the *NH* flow should face toward east and the *HN* displacement vector points toward west, which is contradictory to what is observed in overall displacement angle timeseries. In order to investigate the possible reasons behind such counterintuitive observation, we further analyze the constructed mobility network by separating the trips during one day into four groups. One group consists of the *NH* trips with a positive displacement toward west (Fig. S6.a) and the other group has positive displacement toward east (Fig. S6.b). Trips in the *HN* flow with positive displacement toward west and east are separately depicted in Fig. S6.c and Fig. S6.d respectively. A large proportion of taxi trips with a long journey distance are associated with the trips to or from the O'Hare International Airport and Chicago Midway International Airport. The node associated with the O'Hare

International Airport is classified both as hotspot origin and destination while this is not true for the Chicago Midway International Airport. For the *NH* flow, the O'Hare International Airport has the largest contribution to the overall displacement angle as the average trip length from CBD or the surrounding areas, from which a high proportion of trips originates, to the O'Hare International Airport is much longer compared to the average length of trips from the Midway International Airport to CBD. Therefore, the overall displacement vector tends more toward west for the *NH* flow. However, an opposite pattern is observed for the *HN* flow where a large number of trips start from the O'Hare International Airport and end in CBD, which leads to an overall displacement vector toward west.

Supplementary References

[1] T. Louail *et al.*, "Uncovering the spatial structure of mobility networks," *Nature Communications,* Article vol. 6, p. 6007, 01/21/online 2015.

[2] T. Louail *et al.*, "From mobile phone data to the spatial structure of cities," *Scientific Reports,* Article vol. 4, p. 5276, 06/13/online 2014.

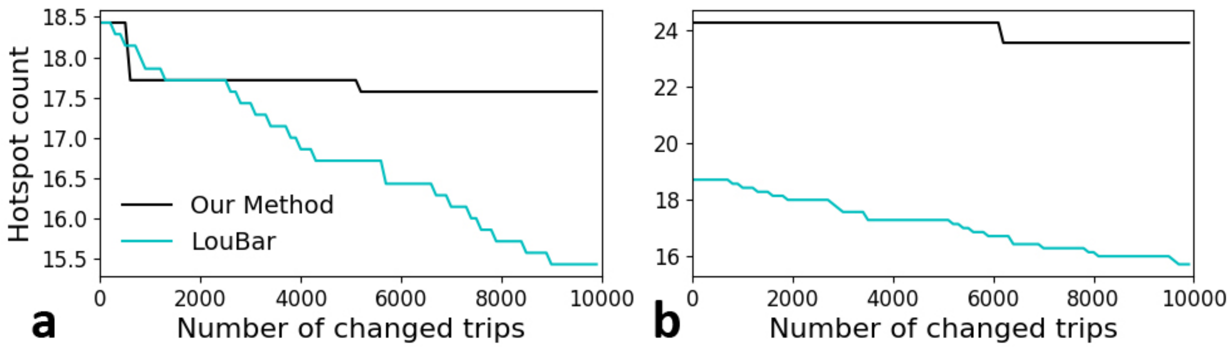

Fig. S1. Number of determined (a) origin and (b) destination hotspots as a function of number of altered trip endpoints; the trips ending in non-hotspot nodes are altered to end at the node with maximum flux. The LouBar method shows much higher sensitivity to the alteration of trip endpoints, although the flux reduction is distributed over a large number of non-hotspot nodes and only the flux of the first ranked node is increasing.

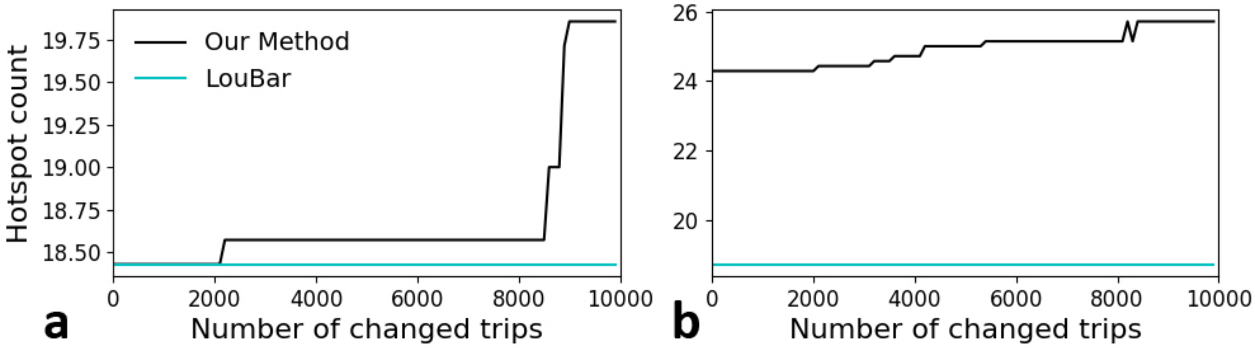

Fig. S2. Number of determined (a) origin and (b) destination hotspots as the trip endpoints to hotspot nodes (excluding the node with highest flux) are reassigned to non-hotspot nodes. The number of hotspots resulted from the LouBar method remains constant even with 10,000 trips manipulated. The change in the endpoints decreases the initial hotspot fluxes and increases the initial non-hotspot fluxes so it is expected that the determined hotspots go through some evolution with increasing number of changes.

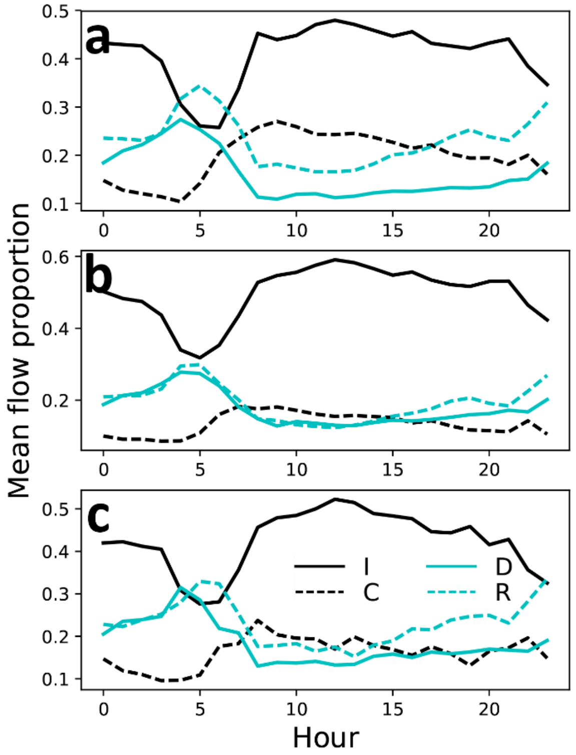

Fig. S3. Hourly mean proportion of flows calculated based on initial OD matrices generated using grids of square cells with edge length of (a) 0.5 km, (b) 1 km, and (c) 2 km.

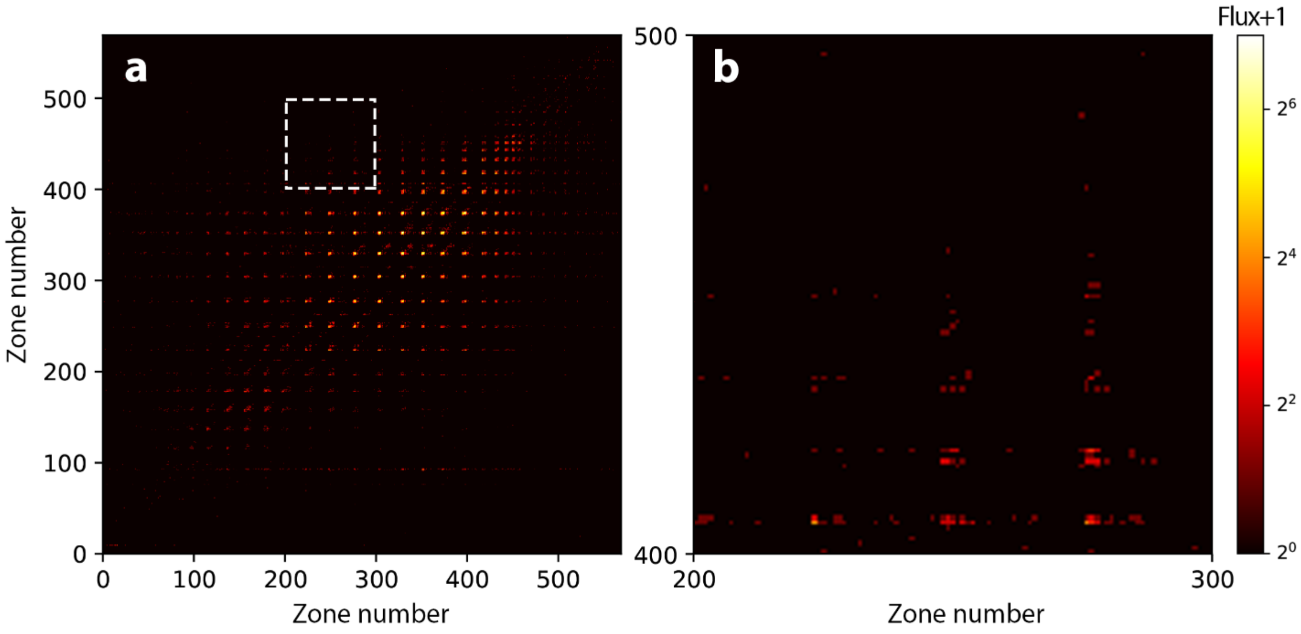

Fig. S4. (a) Original OD matrix for taxi mobility system in New York during hour, generated using grids of square cells with edge length of 1 km. Size of the matrix is 569×569 and there are 5889 non-zero entries in the matrix, meaning that there are 5889 directed links in the corresponding network. The color of each cell represents the number of trips in the corresponding OD demand matrix. The number of trips between zones are scaled logarithmically for better visualization but before scaling we added one unit to their original numbers; the number corresponding to each cell's color is one more than the number of trips between the associated nodes. (b) Details of the OD demand matrix demonstrated by magnifying a subset of matrix entries marked by a white dashed box on the full OD matrix representation.

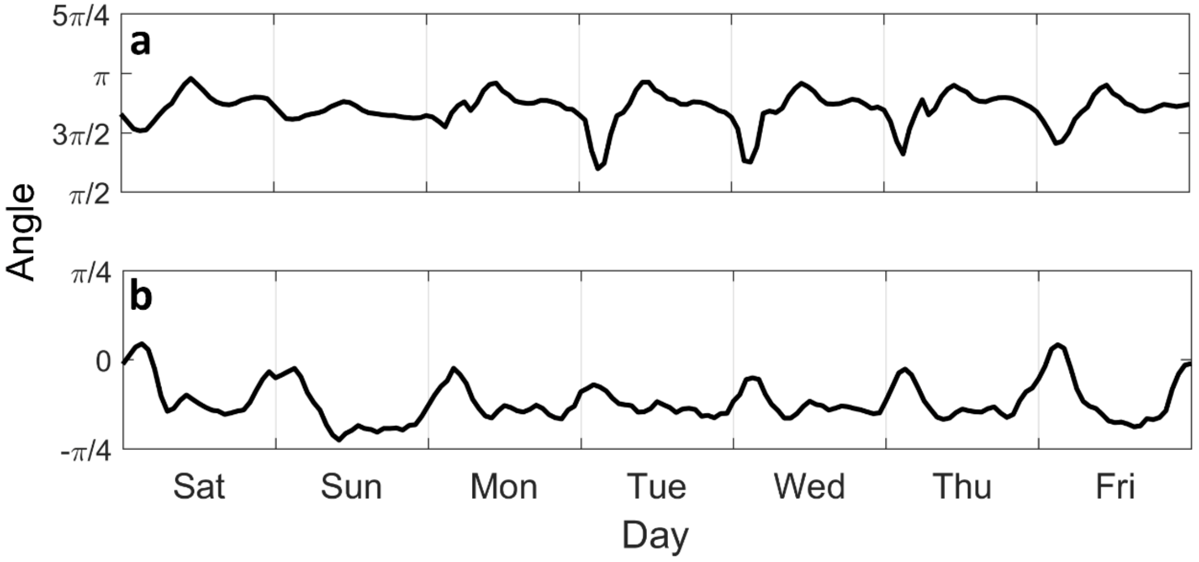

Fig. S5. Angle time series for overall displacement vector of (a) *NH* and (b) *HN* flows in New York. The hourly values angle values are depicted over the last week of February 2015.

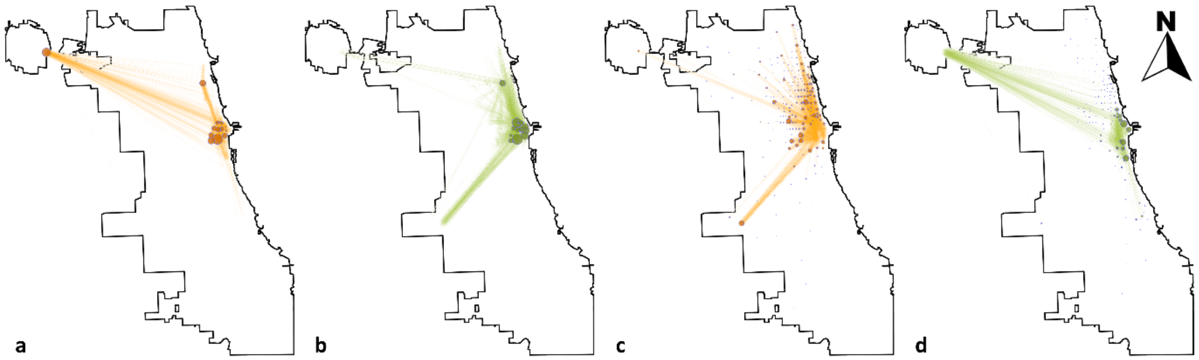

Fig. S6. Chicago taxi trips during a typical weekday. Each straight line connects pick-up and drop-off location of a single trip. (a) *NH* trips larger than zero displacement toward west, (b) *NH* trips larger than zero displacement toward east, (c) *HN* trips larger than zero displacement toward west, and (d) *HN* trips larger than zero displacement toward east. Points show the relative number of drop-offs at in a certain area with their size.
